# PHLPP1 is a metastasis suppressor in melanoma

**DOI:** 10.18632/aging.101544

**Published:** 2018-09-05

**Authors:** Meng Dai, Yanlin Yu

**Affiliations:** 1 Laboratory of Cancer Biology and Genetics, Center for Cancer Research, National Cancer Institute, National Institutes of Health, Bethesda, MD 20892, USA

**Keywords:** PHLPP1, AKT2, AKT3, metastasis suppressor, melanoma

Cancer arises when homeostasis in a susceptible cell is disrupted as a result of the mutation, upregulation and/or downregulation of critical genes. This gene dysregulation bestows upon affected cells such properties as independence from growth control and the ability to migrate to distant sites and grow there, which gives rise to metastasis. PHLPP1 is a member of the pleckstrin homology (PH) domain leucine-rich repeat protein phosphatase (PHLPP) family, which maintains the balanced level of protein (e.g., AKT and PKC) dephosphorization necessary to control a broad range of physiological processes, including cell proliferation, motility, migration, and survival. Changes in the level PHLPP1 or its mutation correlates with many pathophysiological processes, including the development and progression of cancer [1]. For example, PHLPP1 inactivation has been detected in colon, breast, prostate and pancreatic cancers, as well as lymphoma, glioblastoma and melanoma. Whereas overexpression of PHLPP1 induces tumor cell apoptosis and blocks tumor cell proliferation, PHLPP1 knockdown reduces tumor cell apoptosis and promotes tumor cell proliferation [1]. PHLPP1 thus appears to be a bona fide tumor suppressor.

In a comparative study of high- and low-grade prostate cancers, Hellwinkel et al. found that levels of PHLPP1 transcription are significantly decreased in aggressive high-grade tumors [2], which suggests reduction of PHLPP1 may play a role in promoting prostate cancer progression. Consistent with that idea, a study of 218 prostate cancer samples showed that PHLPP (PHLPP1 or PHLPP2) is downregulated in 37% of metastases but in only 11% of primary prostate cancers [3]. It was also recently observed in an animal model of prostate cancer that PHLPP1 deficiency cooperates with PTEN deletion to promote metastasis [4]. In addition, a recent study by Li et al. [5] showed that PHLPP1 inhibits epithelial-mesenchymal transition (EMT) and cell migration and that its deletion promotes invasive tumor growth in Apc^min^ mice, which further confirms that PHLPP1 loss-of-function enhances tumor progression to metastasis. Clinically, analysis of 202 consecutive patients with gastric cancer showed that PHLPP1 expression correlates inversely with lymph node metastasis and that PHLPP1-negative patients had significantly shorter overall and relapse−free survival than PHLPP1−positive patients (both p < 0.001) [6]. In aggregate, these data strongly suggest PHLPP1 acts as an important negative regulator of tumor metastasis. However, PHLPP1’s precise mechanism of action in tumor metastasis has not been elucidated, though we recently demonstrated that PHLPP1 suppresses melanoma metastasis through its phosphatase activity [7].

Cutaneous malignant melanoma is a genetically complex and highly aggressive disease, notorious for both its propensity for metastasis and its poor response to currently available therapy. Increasing evidence indicates that PI3K/AKT pathway activation acts as a driving force for melanoma metastasis. Within that context, we analyzed expression of PHLPP family proteins in poorly and highly metastatic melanoma cell lines and in primary and metastatic melanomas. Our findings revealed that PHLPP1 expression is significantly downregulated or lost in melanoma, and that PHLPP1 deficiency correlates with metastatic potential. Moreover, mutation of PHLPP1 in cutaneous melanoma (SKCM-TCGA) patients correlated significantly with poorer survival [7]. Standard assays of experimental and spontaneous metastasis demonstrated that the ability of highly metastatic melanomas to metastasize is lost or reduced in mouse models overexpressing PHLPP1. Conversely, downregulation of PHLPP1 in poorly metastatic melanomas enhanced their pulmonary metastasis, again suggesting PHLPP1 is able to inhibit melanoma metastasis [7]. That ability of PHLPP1 to suppress metastasis appears to be dependent on its function as a Ser/Thr-specific phosphatase [1], as metastasis is enhanced in melanoma cells expressing a phosphatase-deficient PHLPP1 form but is suppressed in those expressing a PH-deficient form that retained its phosphatase domain [7].

Given that PHLPP1 dephosphorylates serine 474 of AKT2 and serine 472 of AKT3, thereby controlling PI3K/AKT kinase signaling [1], we tested whether PHLPP1 suppresses metastasis through dephosphorization of AKT proteins. Consistent with earlier reports, we found that overexpression of PHLPP1 diminished levels of AKT2 and AKT3 phosphorylation, while shRNA-induced PHLPP1 knockdown had the opposite effect, and a phosphatase-dead PHLPP1 mutant had no ability to reduce phosphorylation of AKT2 and AKT3. Moreover, by introducing constitutively activated AKT1, AKT2 or AKT3 into melanoma cells, we showed that both AKT2 and AKT3 have the ability to promote melanoma metastasis, whereas AKT2 or AKT3 deletion using CRISPR/cas9 or shRNA-induced knockdown blocked melanoma metastasis. Earlier work had already suggested that AKT3 plays a role in melanogenesis and that AKT2 promotes metastasis in several cancers. Our observation showed for the first time that both AKT2 and AKT3 promote the metastasis of melanoma. Notably, our observation that constitutive activation of AKT2 and AKT3 abrogates PHLPP1-mediated inhibition and restores metastatic potential further confirms that PHLPP1-induced metastasis suppression reflects its capacity to selectively dephosphorylate AKT2 and AKT3 [1,7]. These findings raise the possibility that PHLPP1 status could serve as a prognostic marker of melanoma progression and suggest that the PHLPP1/AKT2 axis provides new targets for the treatment of metastatic melanoma.
